# Current Trends in the Management of Temporomandibular Joint Dysfunction: A Review

**DOI:** 10.7759/cureus.29314

**Published:** 2022-09-19

**Authors:** Om C Wadhokar, Deepali S Patil

**Affiliations:** 1 Department of Musculoskeletal Physiotherapy, Ravi Nair Physiotherapy College, Datta Meghe Institute of Medical Sciences, Wardha, IND

**Keywords:** review, cognitive behavioural therapy, pharmacotherapy, physical therapy, dental therapies, temporomandibular joint disorder, temporomandibular joint

## Abstract

The temporomandibular joint (TMJ) is a synovial bi-condylar joint with 3 degrees of freedom. One-third of the adult population reportedly suffers from temporomandibular joint dysfunction (TMD). Females are more commonly affected than males. Almost 50% of TMD patients do not require any intervention, and the symptoms are self-limiting within one year after the onset; however, 75-80% of adults suffering from TMD require medical intervention and it takes up to three years for the complete remission of the symptoms. The clinical features of TMD are clenching, clicking, and locking of the jaw, and occlusion due to faulty posture. Based on the diagnostic criteria for temporomandibular disorder (DC/TMD) criteria proposed in the year 2014, the classification of TMD is done based on axis I and axis II diagnoses. This review aims to provide an overview of TMD and examine available treatment strategies for TMD. Various conservative treatment methods have been proven to be effective, including self-care strategies, dental treatment strategies, pharmacological treatment, physical therapy modalities, manual mobilization, electrotherapy and dry needling, relaxation techniques, intra-articular injections, cognitive behavioral therapy, and surgical corrections.

## Introduction and background

Temporomandibular joint disorder (TMD) is a broad term encompassing various problems associated with the temporomandibular joint (TMJ). About one-third of the adult population suffers from one or more symptoms of TMD [1]. The standard clinical features of TMD include pain in the TMJ and surrounding tissues, which leads to the functional limitation of the Joint [2]. Most adults presenting with TMDs are self-remitting, while some require conservative interventions such as physical therapy and medications [3]. The contributing factors for TMD include teeth clenching, muscular pain, and occlusion of the TMJ due to faulty posture, which predispose individuals to TMD in the long term [4]. TMJ is a synovial joint with excellent mobility and stability. The articular surfaces maxilla and mandible are covered with a fibrous connective tissue. There is a joint disk between the articular surfaces. The mobility to the TMJ is provided by the articular disk, which enables us to perform all the activities of daily living such as speaking, swallowing, and chewing effortlessly [5,6].

Orofacial pain refers to pain over the face, oral cavity, TMJ, and soft tissues, which is a significant cause of non-odontic orofacial pain [7]. The TMD significantly impacts the individual's physical and psychological well-being [8]. TMD treatment can involve high economic costs leading to depression and psychological affliction [9,10].

Prior to 2000, clinicians considered malocclusions to be the leading cause of TMD; later on, in early 1990, it was found that role of malocclusion in TMD was minimal [11]. Surgical procedures for TMDs increased significantly in 2010, but due to a lack of evidence on arthrocentesis for TMD, the use of operative procedures has reduced significantly [12]. In recent years, bio-psychosocial models have been used. This model includes physical, psychological, and pharmacological therapies [13].

## Review

Methodology

An extensive search was conducted on databases like Pubmed, Scopus, Web of Science, Cochrane, Medline, and Embase using keywords such as "Temporomandibular Joint", Temporomandibular Joint Dysfunction", "Pain", "Dental Therapies", "Physical Therapy", "Pharmacotherapy", "Surgical Intervention"; relevant articles published till may 2022 were included in the study

Classification of Temporomandibular Joint Disorder

The research diagnostic criteria for TMD (RDC/TMD) were used for diagnosing and grading TMD. The RCD/TMD is based on the bio-behavioral pain model, which consists of two axes. Axis I denotes physical signs and symptoms such as painful myofascial disorders, disc sub-luxation, and arthritis, while axis II consists of psychological and disability factors [14]. In the most recent version of RDC/TMD, new criteria were published, named DC/TMD, a more comprehensive instrument for both axes for researchers and clinicians [15]. Neurophysiology of trigeminal sensory system: the fifth cranial nerve is considered a mixed cranial nerve with three branches: (a) ophthalmic, (b) maxillary branch, and (c) mandibular nerve. The craniofacial innervation and nerve signals are similar throughout the body; craniofacial innervations have some peculiarities as they depend on several anatomical and functional structures of primary afferent neurons emerging from the trigeminal ganglion. On the other hand, cranial peripheral nerves have fewer efferent sympathetic axons than spinal nerves. Some researchers have postulated that this peculiarity could have a relative influence on the painful state maintained by the sympathetic nervous system itself in the trigeminal region. Other sympathetic differences between the trigeminal area and the rest of the body are related to the intra-cranial and cutaneous blood vessels. In the trigeminal area, these vessels receive both parasympathetic and sympathetic innervations; however, in the segmental levels, parasympathetic innervation is infrequent or nonexistent. Pathophysiology of TMD: TMD pain is caused mainly by peripheral mechanisms and a poor correlation between TMD pain and tissue pathology. This has led to the conclusion that some patients may have altered central nervous system pain processing, which is characterized by the heritable gene. The psychological stressor contributes to the pain from masticatory muscles. TMD pathology is associated with gender, and females are more commonly affected by TMD than males. One study suggests that the increased prevalence of TMD in females is due to estrogen [16].

Etiology of Temporomandibular Joint Disorders

The etiology of the TMD is not clearly known. Still, it is proposed to be multi-factorial, including abnormal occlusion, bruxism, teeth grinding, lip biting, stress, anxiety, joint capsule inflammation, muscle spasm, and abnormalities in the intraarticular disk; dental occlusion is a frequent presentation in individuals diagnosed with TMD [11,17]. Evidence has linked stress, anxiety, and emotional instability more specifically with individuals with pain. Of note, 75% of individuals with TMD have a significant psychological problem [18].

Diagnosis of Temporomandibular Joint Disorder

Pain, reduced jaw mobility, headache, neck pain or stiffness, teeth grinding, and pain with mouth opening are some of the symptoms associated with TMD [17]. Physical examination: it involves the examination of masticatory muscles for the presence of spasms or trigger points in the muscles such as masseter, temporalis, and sternomastoid. This is done by careful palpation of the muscles. A joint assessment is performed by placing the fingers over the TMJ and instructing the individual to perform mouth opening as palpate for the TMJ clicking/popping, indicating intra-articular disk displacement. Pain and swelling near TMJ indicate intra-articular inflammation. The cervical posture and head position are assessed by using a plumb line to assess the head posture as the forward head posture leads to dental occlusion and TMD. The markers for effective treatment include reduced pain, improved function, and everyday quality of life [18-20]. Differential diagnosis: 55% of patients referred to a neurologist with chronic headaches were found to have significant signs and symptoms of TMD. TMDs often present with pain and commonly undifferentiated headache [21]. Diagnostic testing: radiologic imaging is one of the available methods to look into TMD. It is practical and should be used in most severe patients with TMD [22].

Treatments for Temporomandibular Joint Disorder

**Figure 1:**
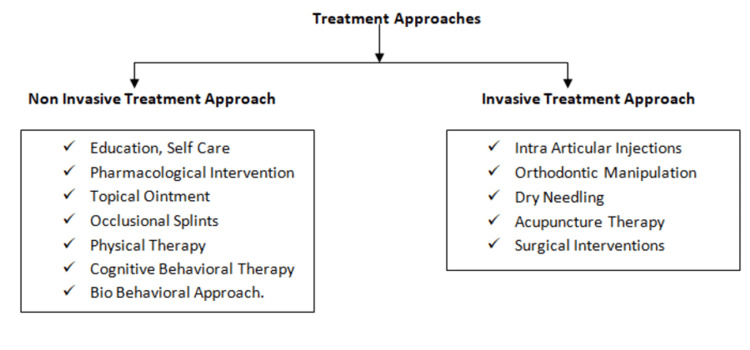
Treatment approaches

The orthodontic and surgical approaches will permanently alter the joint anatomy, and hence they are avoided in several cases, and patients go for conservative therapies such as physical therapy, pharmacotherapy, occlusal splints, behavioral approach, and self-care. Almost 50% of individuals' symptoms remit within one year while 85% of the population suffering from TMD recovers completely in three years [23]. The available treatment approaches are depicted in Figure 1.

The non-invasive treatment measures for TMD include medications, dental therapies, physical therapy, and psychological treatment, which have been shown to improve the overall quality of life of the individual [19].

Education and Self-Care

Self-awareness and education are valuable strategies for pain management. A slight benefit was obtained with education when comparing this intervention with occlusal splints. This intervention approach, when compared with certain interventions like manual therapy and exercise, showed no additional benefits [24,25]. Self-care is the mainstay of the treatment. It includes the prescription of simple exercises, and behavioral modifications are encouraged. This is indicated in a patient with a new or intermediate clinical presentation [18].

Pharmacological Intervention

This intervention includes acetaminophen, and non-steroidal anti-inflammatory drugs, which help with acute and chronic pain; for muscular spasms and teeth-clenching muscles, relaxants are prescribed, such as benzodiazepines. If this approach fails, then tricyclic antidepressants can help with pain and teeth grinding. Antidepressants used for chronic pain can also be prescribed in the case of TMD. Care should be taken while prescribing selective serotonin uptake inhibitors; they might induce bruxism [19,26].

Intra-Articular Injections

The inflammation of the TMJ and the capsule can be resolved with intra-articular injection with local anesthetics or corticosteroids. These injections are only used for severe acute exacerbation and after the failure of conservative treatments. A systematic review found non-significant evidence for repetitive intra-articular injections in TMD. Chronic bruxism and myofascial pain can be treated with local anesthetics and botulinum toxin [27,28]. Out of five studies on the subject, two showed a prominent reduction in pain, one showed an equal reduction in pain with manual therapy, and the other two studies showed no significant reduction in pain with botulinum toxin compared to a placebo. More research is required to assess the long-term effect of BTX on the injected muscles. The research has shown the size of the muscle being recovered, but also demonstrated the loss of the contractile function; after one year of BTX injection and placebo injection for trapezius muscle pain, there was no difference in the pain intensity measurement [29,30].

Dental Therapies

Dental occlusion splints and permanent dental adjustment are the mainstays of the treatment; the splints are selected based on the condition. The occlusal splints are used to correct the alignment of the upper and the lower teeth, and the non-occluding splints are mainly used for mouth opening, releasing muscle tension, and grinding of teeth. The cost of non-occluding splints is high, and hence very few can afford the splint. Permanent occlusion adjustments are made by professional orthodontics [28,31].

Physical Therapy

Physical therapy plays an important role in reducing pain, improving joint mobility, restoring motor functions, and reducing inflammation to relieve the symptoms of TMD. The intervention includes several types of exercise [32,33]. Rocabado exercises: in these, the patient is instructed to put the tongue on the roof of the mouth and take six deep breaths followed by a sequence of exercises. Goldfish exercises: in this exercise, the individual is asked to put his tongue to the roof of the mouth and then place one index finger on the TMJ while the other on the chin. Range of motion exercises: this is done to relieve stiffness and improve symptoms [34,35]. Joint mobilization: the joint restriction can be improved by manual joint mobilization by placing both the thumb on the molars, gently mobilizing the mandible for pain relief, and improving the range of motion. Soft tissue mobilization: it has proven to be effective in reducing muscular pain by gently mobilizing the soft tissue and applying gentle pressure over the area of the spam trigger point [36-39].

Electrotherapy for Pain Modulation

Transcutaneous electrical nerve stimulation (TENS): it is applied to reduce pain by using mild electrical current. Both the electrodes are applied on the TMJ, and the secured parameters of the TENS are set; the frequency is set to 50-100Hz, which is of low intensity, and the pulse width is between 50-200 microseconds. Low-level laser therapy (LLLT): this is used by physiotherapists for various musculoskeletal conditions. It is a non-invasive treatment modality that generates single-wavelength light. It emits no heat, sound, or vibrations LLLT is also known as photobiology or biostimulation. It accelerates connective tissue repair and acts as an anti-inflammatory agent. The wavelength used for treatment lies between 632 to 904 nm. Therapeutic ultrasound: it is a sound wave that requires a medium to propagate. The frequencies used are 1 MHz and 3 MHz. The velocity of the ultrasound depends on the medium used for the coupling; the speed of ultrasound from most of the tissue is the same as the velocity of ultrasound through saline. It has both thermal and non-thermal effects. The non-thermal effect includes cavitations, acoustic streaming, and micro-massage ultrasound for inflammation, proliferation, and remodeling of the tissue, which have been proven to be effective in alleviating pain and improving the functionality of the individuals [40,40,41]. Relaxation technique: this includes trigger point massage, jaw relaxation, chin tuck, and breathing exercises. Dry needling and acupuncture therapy: A few studies have shown that dry needling is effective in relieving pain in TMD. It has been proven to be effective in reducing the referred pain, myogenic pain, and trigger point [42-45]. A few studies have shown that the effect of dry needling is the same as the effect obtained after the injection of lidocaine and corticosteroid [46]. Acupuncture is an excellent therapeutic approach for pain relief in acute conditions like myofascial TMD but is contraindicated in conditions with temporomandibular joint restriction. The exact mechanism for the pain relief behind acupuncture is not yet clearly known. Still, it is hypothesized to be due to the release of spinal and supraspinal serotonin, endogenous opioids, and other transmitters with anti-inflammatory actions. Acupuncture is applied after the identification of the area of the pain [47,48].

Cognitive Behavioral Therapy

It is one treatment approach available for patients suffering from TMD. This intervention, along with other conservative approaches mentioned above, helps to manage the thoughts, feelings, and behavior that aggravate the symptoms It plays a significant role in conjunction with other therapies treating the psychological causes of the pain [43].

Bio-Behavioral Approach

The bio-behavioral approach for diagnosing and treating the patient with chronic TMD has shown that psychological factors play an essential role along with pain history, current emotional and cognitive status beliefs, learned behavior, and tackling strategies. This approach allows the patient to acquire the ability to self-manage, leading to an improvement in overall functioning. Based on the recent clinical findings, the assessment, diagnosis, and treatment of TMD patients require a multidimensional approach, which the bio-behavioral approach provides. This model is designed for musculoskeletal disorders. This approach is based on the following objectives: (a) reduction of pain perception, (b) improvement of motor behavior, and (c) improvement of the cognitive and emotional factors related to the experience of pain [49].

Surgical Interventions

In various cases such as internal derangement, degenerative changes, and joint pathology, the arthrocentesis/arthroscopic approach is used. In the case of internal derangement, surgical intervention is not recommended, and a conservative approach and rehabilitation are recommended instead. For other conditions like disk displacement, minimally invasive intervention is used. Recent evidence has shown that the injection of platelet-rich plasma and arthrocentesis is effective in osteoarthritis of TMJ; this should be confirmed by conducting more studies. A summary of the literature review is presented in Table 1.

**Table 1:** Review of studies

Author	Study type	Outcome	Intervention	Study duration	Sample population	Result	Analysis
Rhodes et al [38]	Review	-		-	Studies on physical activity	Regular physical activity in the prevention of at least 25 medical conditions	Physical activity plays an important role and is clinically relevant for the health status
Chellappa et al. [39]	Randomized controlled trial	NPDS (numeric pain distress scale). Mandible active range of motion	Transcutaneous electrical nerve stimulation, low-level laser therapy	6 consecutive weeks	Temporomandibular joint dysfunction	Low-level laser therapy was more effective than transcutaneous electrical nerve stimulation	Individuals with temporomandibular joint dysfunction shall be treated with Low-level laser therapy for more efficient results
Sakurai et al. [40]	Interventional study	Crevier fluid, bacterial measures	Low-level laser therapy	-	People showing gingival inflammation	Low-level laser therapy inhibits prostaglandin E_2_ by the level of personality functioning in the hepatocytic growth factor	Low-level laser therapy has therapeutic benefits for gingivitis and periodontitis
Aggarwal et. al. [41]	Review article	Long-term pain intensity, depression	Psychosocial intervention, cognitive behavioral therapy	Articles of the year 2020	Chronic orofacial pain	Weak evidence to support the use of psychosocial intervention	The noninvasive nature of treatment is preferred as it does not affect the anatomical structures
Kotiranta et al. [42]	Systematic review	Multi-dimensional pain inventory	Tailored temporomandibular joint dysfunction treatment, conventional temporomandibular joint dysfunction treatment	Articles in March 2013	Temporomandibular joint dysfunction articles	In all the trials, the result supports that tailored treatment is more effective	A well-tailored treatment regimen shows a great response
Fernández-Carnero et al. [43]	Randomized controlled trial	Pressure pain threshold, active jaw opening	Sham dry needling, deep dry needling	2 days	Patients diagnosed with temporomandibular joint dysfunction	Subjects showed greater improvement with deep dry needling	Deep dry needling helps in the treatment of active trigger points with significant improvement in jaw opening and pain
Blasco-Bonora et al. [44]	Case series	Pressure pain threshold, active jaw opening	Deep dry needling	1 week	Temporomandibular joint dysfunction related to disability to sleep bruxism	Significant improvement in pain threshold and active jaw opening post deep dry needling	Temporomandibular joint dysfunction and sleep bruxism are associated with immediate and 1- week relief in pain and jaw opening
Venâncio Rde et al. [45]	Randomized controlled trial	Pain intensity	Dry needling, lidocaine, lidocaine-associated with corticoids	12 weeks	Patient with myofascial pain	Post-injection sensitivity association of lidocaine with corticoids showed the best results	Lidocaine with corticoids injection shows the best results
Goddard et al. [46]	Clinical trial	Visual analog scale	Dry needling	-	Masseter muscle pain	Both acupuncture and sham acupuncture reduced the pain of masseter	Both treatments can be used for efficient clinical results
Sekido et al. [47]	Comparative study	Paw pain threshold	Electro acupuncture		Acute inflammatory pain in rats	It is like the electroacupuncture analgesia in the rats with carrageenan-induced inflammation differing from normal rats	Peripheral opioid receptors, electroacupuncture analgesia in acute inflammation condition
Le Pera et al. [48]	Relational study	Pain-related psychological questionnaire	Conditioned pain modulation, pain temporal summation, transcranial magnetic stimulation neurophysiological assessment		45 healthy subjects	This increased corticospinal excitability of the primary motor cortex is associated with inhibitory pain modulation as assessed by condition pain modulation	Condition pain modulation is an objective and easy-to-measure parameter to allow for better individual assessment of the pain modulation profile

## Conclusions

We conclude that like other musculoskeletal disorders, TMD, which also has a muscular origin, can be managed by the above-mentioned treatment modalities. One-third of the adult population suffers from TMD and it drastically affects the quality of life of the individual; previous studies suggest that no single treatment modality is efficient in reducing the pain but a multidisciplinary approach is required for the complete remission of the symptoms. Conservative management is recommended for most TMD patients, which includes education, self-care, dental therapy, occlusal splints, intra-articular injections, topical ointments, pharmacotherapy, physical therapy, dry needling, acupuncture therapy, electrotherapy, cognitive behavioral therapy, psychological treatment, and bio-behavioral approach. When all the conservative methods fail to reduce the symptoms, then surgical intervention is used. In cases of surgical corrections of the joint structure or soft tissue, after the surgical procedure, individuals should continue physical therapy for complete restoration of the function and return to normal lives. Pain modulation in TMD can be effectively achieved by pain medication, soft tissue mobilization, grade I and grade II joint mobilization, and electrotherapy modalities such as TENS and ultrasound.
